# Logic model-based performance management systems for export promotion agencies

**DOI:** 10.12688/openreseurope.20545.2

**Published:** 2025-09-23

**Authors:** Stéphane Ruiz-Coupeau, Juan Manuel Ramon-Jeronimo, Raquel Florez-Lopez

**Affiliations:** 1 Department of Accounting and Financial Economics, University of Seville, Seville, Andalusia, 41004, Spain; 2 Department of Financial Economics and Accounting, Pablo de Olavide University, Seville, Andalusia, 41013, Spain

**Keywords:** Performance Management Systems, Logic Model, Export Promotion Agencies, Accountability

## Abstract

**Background:**

Export Promotion Agencies (EPAs) play a crucial role in facilitating the internationalization of small and medium enterprises (SMEs), contributing significantly to economic growth. The complexity of their operations, diversity of services provided, and challenges in attributing outcomes complicate the effective implementation of Performance Management Systems (PMSs).

**Method:**

This study investigates how the Logic Model framework can be utilized as a strategic, structured, and flexible tool to design and implement PMSs tailored to the unique operational needs of EPAs. A qualitative, exploratory multiple-case study approach was applied, involving various types of EPAs within Spain—including provincial, regional, national, and a regional EPA belonging to a European network.

**Results:**

The research identifies significant variability in PMS sophistication and logic model adoption across agencies. The Logic Model, emphasizing causal linkages between inputs, activities, outputs, and outcomes, offers EPAs a coherent framework to enhance transparency, accountability, and strategic learning. An extended illustrative case of a European-networked regional EPA highlights best practices in integrating structured client journeys, performance monitoring, and dynamic feedback mechanisms into service delivery.

**Conclusion:**

This research proposes a comprehensive methodology for implementing Logic Model-based PMSs within EPAs, emphasizing stakeholder participation, flexible indicators, systematic service quality evaluation, and continuous adaptive management. By addressing the identified gaps in performance management practices within EPAs, the study contributes to both academic literature and practical guidelines, ultimately supporting the international competitiveness of SMEs and economic development objectives.

## Plain language summary

Export Promotion Agencies (EPAs) support small and medium-sized enterprises (SMEs) in expanding to international markets, which contributes to economic growth. However, evaluating the effectiveness of their diverse services can be challenging. This study examines how the Logic Model -a graphic illustration of the relationship between a program's resources, activities, and its intended- effects- can serve as a useful tool to help EPAs design and manage their activities more clearly and effectively. Based on case studies of several EPAs in Spain, the research finds that using the Logic Model improves the ability to plan, monitor, and demonstrate results. It helps agencies better connect their resources, services, and intended outcomes, leading to greater transparency and continuous improvement. A detailed example of a successful EPA shows how this approach can enhance support for SMEs and improve overall agency performance.

## Introduction

EPAs have become central actors in contemporary public policy, aiming to facilitate the internationalization of domestic firms, enhance national competitiveness, and stimulate economic growth ( Seringhaus & Rosson, 2012). Their mission is particularly critical for SMEs, which often face significant barriers to entering and expanding in foreign markets ( Cruz *et al.*, 2018). These barriers include information asymmetries, market failures, and transaction costs that inhibit the optimal allocation of resources for internationalization efforts, even among firms possessing sufficient internal capabilities ( Aalto & Gustafsson, 2020; Cruz, 2014).

Although the evaluation of Government Export Promotion Programs (GEPPs) has been an active area of academic inquiry for decades, the evidence regarding their effectiveness remains mixed ( Freixanet, 2022; Kahiya, 2024; Ribeiro & Forte, 2019). Researchers have pointed to inconsistencies in methodologies, differences in the selection of performance indicators, and difficulties in isolating program impacts from external factors as reasons for these inconclusive findings. Much of the literature focuses on final outcomes, such as export sales increases, while paying less attention to intermediate results or the organizational processes that drive them ( Alvarez, 2004; Rose, 2007; Zou *et al.*, 2003).

Performance monitoring and evaluation of public sector programs should not be limited to ex-post assessments; rather, it must be conceived as an ongoing process that begins during the planning phase (program inception/ex-ante evaluation), continues during implementation (program operation/in itinere evaluation), and concludes with verification of results and impacts (program evaluation and dissemination/ex-post evaluation) ( Savaya & Waysman, 2005). This continuous performance management allows organizations to monitor activities, learn from their experiences, adjust strategies in real-time, and strengthen accountability mechanisms internally and externally ( Hatry, 2006; McLaughlin & Jordan, 2015; Poister, 2015).

However, designing PMSs for public organizations—and especially for EPAs—presents unique challenges ( Freixanet, 2022; Poister, 2015). These organizations operate in complex, multi-stakeholder environments where outcomes are influenced by external variables such as global economic trends, regulatory changes, and firm-specific characteristics ( Kahiya, 2024). Additionally, the long-term nature of export development processes complicates the measurement of immediate results ( Freixanet, 2022).

To address these challenges, the Logic Model framework has gained traction among practitioners and scholars as a tool for conceptualizing, planning, and evaluating public programs ( Coryn *et al.*, 2010; Hatry, 2006; McLaughlin & Jordan, 2015). The Logic Model, by articulating causal linkages and structuring program (and related services) logic, offers an appropriate methodological foundation for developing PMSs in EPAs. It provides a roadmap for designing interventions, selecting indicators, monitoring progress, and evaluating results, all while maintaining the flexibility needed to adapt to changing circumstances ( Hatry, 2006).

Despite its widespread application in other fields of public policy—such as health ( Smith *et al.*, 2020), innovation policy ( Jordan, 2010) and development cooperation ( Ringhofer & Kohlweg, 2019) academic research on the use of Logic Models to design PMS within the context of export promotion agencies remains scarce ( Seringhaus, 1990). The peculiarities of EPAs, including the diversity of services offered, the heterogeneity of their client base, and the multiplicity of external influences, require a complex use of the Logic Model.

This paper seeks to fill this gap by proposing the Logic Model as a structured, flexible, and stakeholder-centric tool for the development of PMSs in EPAs. Based on a qualitative, exploratory multi-case study methodology, and illustrated through an extended analysis of a regional EPA belonging to a network funded by the European Commission, this study aims to answer to following research questions:

(1) *How are Performance Management Systems designed in Export Promotion Agencies?*
(2) *How can PMSs be particularised to the needs and realities of EPAs using the Logic Model framework?*

And proposes a structured methodology for designing and implementing PMSs in EPAs that enhance strategic learning, accountability, and service effectiveness.

By contributing to the academic literature on public performance management and offering practical guidance to policymakers and practitioners, this study aspires to support the continuous improvement of export promotion strategies and, ultimately, the competitiveness of SMEs in global markets.

## Theoretical background

### Public Performance Management Systems

Public sector organizations have increasingly embraced PMSs to enhance operational efficiency, guide decision-making, and reinforce accountability mechanisms ( Placek *et al.*, 2021; Poister, 2015). The evolution of performance management in the public sector can be broadly divided into three distinct eras, each of which has contributed to shaping contemporary PMS approaches ( Kline & Aristigueta, 2017; Pollitt & Bouckaert, 2011).

During the Scientific Management Era (early 20th century to the 1960s), rational planning and technical expertise were emphasized. Tools such as Cost-Benefit Analysis (CBA) and Cost-Effectiveness Analysis (CEA) emerged to guide resource allocation, although practical difficulties in quantifying social benefits and costs often limited their application ( Cellini & Kee, 2010).

The New Public Management Era (1970s–1990s) brought about a paradigm shift, inspired by private sector management techniques. It introduced market mechanisms into public administration and emphasized outcomes rather than processes. Strategic planning tools such as the Balanced Scorecard ( Kaplan & Norton, 1992) gained traction. Alongside, methodologies such as Lean Six Sigma ( Tjahjono *et al.*, 2010) and performance-based budgeting practices ( Mauro *et al.*, 2021) were introduced to foster efficiency and customer orientation.

From the 1990s onward, the New Public Governance Era highlighted the importance of networks, collaboration, and stakeholder participation in the delivery of public services. The focus shifted towards achieving societal value through partnerships, adaptive management, and citizen engagement ( Pollitt & Bouckaert, 2011). The Logic Model can be seen as a response to performance challenges in the public sector, since it provides an explicit structure linking strategic objectives, activities, outputs, and outcomes. In this way, it operationalizes what Johnsen *et al.* (2024) identify as the key role of PMS design: bridging strategy formulation and the effective use of performance information

**Figure 1.**
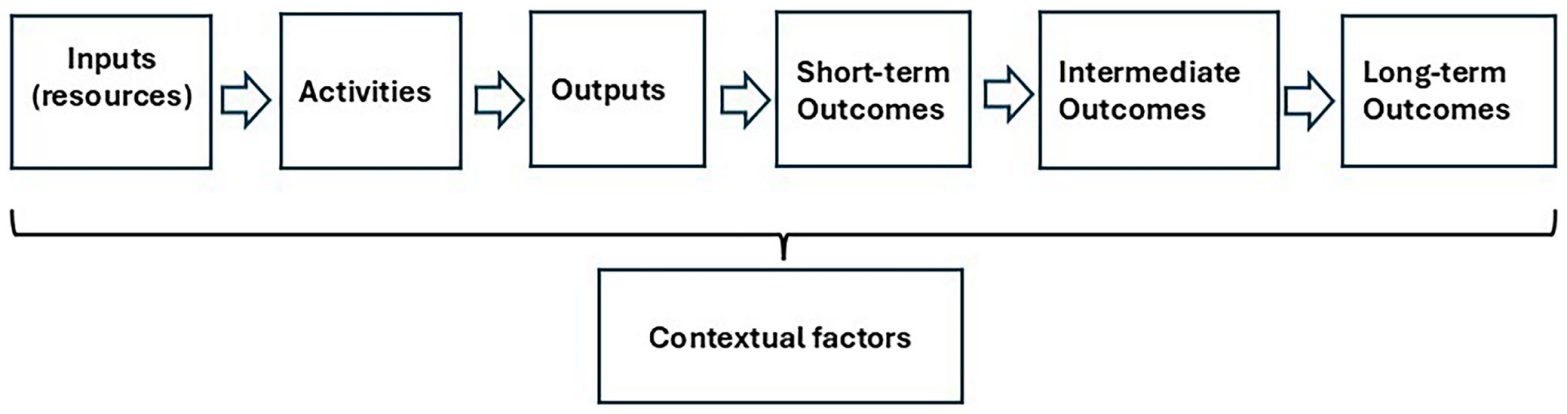
The Logic Model framework. Source: the authors based on Harty (2006).

Across these eras, the Logic Model framework ( Figure 1) has become an increasingly important tool for structuring and evaluating public programs. Logic Models offer a systematic way to articulate the links between program inputs (resources), activities, outputs, and different levels of outcomes. They make assumptions and causal pathways explicit, thus facilitating a clearer understanding of how interventions are expected to generate desired changes. In addition, external contextual conditions, beyond the direct control of the program, can shape its effectiveness in either supportive or constraining ways ( Hatry, 2006; McLaughlin & Jordan, 2015).

While the Logic Model is a widely used tool for planning and evaluating programs, it has several limitations. It tends to assume a linear cause-effect relationship, which oversimplifies the complex and dynamic nature of real-world interventions ( Funnell & Rogers, 2011). The model often struggles to capture intangible or emergent outcomes ( Rogers, 2008). Additionally, it can lead to a focus on short-term outputs rather than long-term impacts, and it is not inherently adaptive to program changes over time ( Hatry, 2006).

It is important to distinguish between performance measurement—the process of collecting data about program activities and outputs—and performance management, which uses that data to inform strategic decision-making and continuous improvement ( Lebas, 1995; Radnor & Barnes, 2007). A PMS should integrate both dimensions, serving not only accountability requirements but also organizational learning and innovation ( Fryer *et al.*, 2009).

Implementing effective PMSs in public sector contexts remains challenging ( Garengo & Sardi, 2021). Organizations must often balance multiple, sometimes conflicting objectives; deal with resource constraints; and operate in complex political and social environments ( Fryer *et al.*, 2009). As a result, tools like the Logic Model, which promote clarity and focus without rigidifying processes, have become increasingly valuable.

### Export promotion agencies and their performance management needs

EPAs are public or quasi-public organizations dedicated to facilitating and supporting the internationalization of domestic firms. Their services typically include providing market intelligence, offering training and consultancy, organizing trade missions, supporting regulatory compliance, and in some cases, providing financial assistance for internationalization activities ( Lederman *et al.*, 2010; Seringhaus & Rosson, 2012).

Unlike many other public programs, EPAs’ interventions aim to stimulate complex processes of firm transformation over extended periods. Export success is often contingent upon a confluence of internal firm capabilities and external environmental factors, making it difficult to attribute outcomes directly to EPAs’ support ( Freixanet, 2012; Freixanet, 2022; Freixanet & Churakova, 2018). Moreover, the diversity of services offered by EPAs ( Leonidou *et al.*, 2011) complicates the design of coherent performance evaluation systems.

Several specific challenges characterize performance management in EPAs ( Freixanet, 2022; Naidu & Rao, 1993; Seringhaus & Botschen, 1991). One major issue is outcome attribution, since export results are influenced by numerous external factors—such as global economic conditions, political events, or firm-specific strategies—that complicate efforts to isolate the agency’s specific contribution. Additionally, there is often a significant time lag between the delivery of services and the realization of their effects; for instance, the benefits of participating in a trade mission may only become evident years later through new business contracts. Another challenge lies in service heterogeneity, as EPAs provide a diverse array of services, from disseminating information to fostering technological collaborations, each of which demands distinct performance indicators. Furthermore, EPAs must navigate stakeholder diversity, balancing the expectations of political authorities who require evidence of effectiveness with the needs of business clients who demand tailored and high-value services.

As the COVID-19 pandemic demonstrated, EPAs must also be resilient and adaptable to sudden shocks. A recent global study with EPAs found that high-income countries increased budgets, adopted recovery plans, and implemented digital programs such as e-commerce training and B2B matching events, whereas low-income countries struggled to adjust. These adaptive responses were positively correlated with export performance ( Choi *et al.*, 2025). This evidence highlights that PMSs for EPAs must not only address efficiency and accountability, but also incorporate adaptability and responsiveness in times of crisis.

Despite the growing body of research analysing the effectiveness of export promotion programs ( Alvarez, 2004; Cansino *et al.*, 2013; Comi & Resmini, 2020; Kahiya, 2024; Malca *et al.*, 2020; Martincus & Carballo, 2010; Ribeiro & Forte, 2019; Rose, 2007), relatively little attention has been paid to how EPAs internally organize their performance monitoring and management systems. Given these complexities, there is a pressing need for multidimensional PMSs. These systems should clarify the causal mechanisms linking agency activities to firm-level and macroeconomic outcomes. They should also capture intermediate results, such as changes in firm capabilities, international market engagement, or network expansion. In addition, PMSs need to integrate efficiency measures, service quality indicators, and client satisfaction data into a holistic performance framework. Finally, they must allow for the consideration of contextual factors and external shocks that affect export performance.

The Logic Model facilitates the visualization of the causal relationships between inputs, activities, outputs, and outcomes. It clarifies program assumptions, articulates theories of change, and supports systematic performance monitoring and strategic adaptation ( Hatry, 2006). By articulating these causal linkages and structuring program logic, the Logic Model offers an appropriate methodological foundation for developing PMSs in EPAs. This makes it a particularly valuable tool for EPAs’ managers seeking to enhance the effectiveness and accountability of their interventions. It provides a roadmap for designing interventions, selecting indicators, monitoring progress, and evaluating results, all while maintaining the flexibility needed to adapt to changing circumstances.

Beyond the internal complexity of their service portfolios, EPAs are also subject to accountability pressures from multiple external actors, including ministries, regional governments, and supranational funders. While these overlapping demands can sometimes be mutually reinforcing, they also frequently generate conflicting expectations and reporting requirements. Such situations increase the risk of decoupling, whereby indicators are collected primarily to satisfy compliance obligations rather than to guide strategic management or organizational learning. Recognizing this tension is essential, as it highlights the need for PMSs that balance external accountability with the agency’s own capacity to adapt and improve. By making causal linkages explicit and co-constructing a shared results logic, the Logic Model offers a pathway to reduce fragmentation and better align performance monitoring with meaningful strategic objectives ( Freixanet & Churakova, 2018; Pollitt & Bouckaert, 2011; Radnor & Barnes, 2007).

Building upon these insights, this study proposes a Logic Model-based approach tailored to the operational realities of EPAs, aiming to enhance their strategic management, learning capacity, and accountability to stakeholders.

## Research methodology

The objective of this study is to explore how EPAs can design and implement PMSs based on the Logic Model framework. Given the relative scarcity of prior research on this specific topic and the need for an in-depth understanding of organizational practices and challenges, a qualitative, exploratory multiple-case study design was chosen as the most appropriate methodological approach ( Yin, 2018).

Qualitative case studies are particularly suitable when the research seeks to investigate complex phenomena within their real-life contexts, where the boundaries between phenomenon and context are not clearly defined. In the case of EPAs, their activities, structures, and performance management practices are deeply influenced by political, institutional, and economic environments, making a qualitative and contextualized approach essential ( Harrison *et al.*, 2017).

### Research design

The research design follows an exploratory multiple-case study methodology. This design allows for both literal replication - where similar results are expected across cases - and theoretical replication -where contrasting results are anticipated for predictable reasons- ( Yin, 2018).

By analysing different types of EPAs—provincial ^1^, regional, national, and a regional EPA belonging to a network funded by the European Commission —the study captures variations in organizational size, resource endowment, strategic orientation, and external accountability pressures. This diversity enhances the robustness of the findings and allows for greater analytical generalization.

In the Spanish context, the landscape of EPAs is multi-layered, combining provincial, regional, national, and European network levels. At the provincial level, chambers of commerce typically operate as public-law corporations with modest staff complements (often fewer than 20 employees dedicated to internationalization), focusing on training, advisory, and documentation services for SMEs. Spain has 83 territorial chambers of commerce covering the whole country. Regional EPAs represent larger organizations under the authority of the autonomous regions (Spain has 17), generally employing several hundred staff across headquarters and a network of foreign offices, which allows them to provide sector-specific training, trade fair participation, and in-market facilitation. At the national level, the state export promotion agency oversees a broad portfolio of programs with a workforce exceeding 1,000 employees and operates a global network of more than 104 trade offices, providing financing instruments alongside information and training services. Finally, some regional EPAs act as nodes within pan-European networks where they combine regional specialization with EU-mandated service standards and reporting. Across these institutional levels, the main categories of performance indicators tend to cover outputs (e.g., number of firms trained or missions organized) and satisfaction surveys. A more detailed description of each agency and the classification of its services under the four categories defined by Leonidou *et al.* (2011) can be found in Annex 1.

The case study protocol encompassed the definition of the study's research questions, the identification of criteria for selecting cases, the development of a semi-structured interview guide, and the triangulation of findings through the analysis of public external data. This systematic approach ensured consistency across cases and enhanced the validity and reliability of the research.

### Case selection

Four cases and five interviews were purposively selected to represent the different types of EPAs mentioned within Spain (see Table 1):

**Table 1.** Profile of the interviewees.

Interview ID	Role	Case
Int1	Director of Foreign Trade Department at a Provincial Chamber of Commerce	Provincial EPA
Int2	Director of the Foreign Offices Network of a Regional EPA	Regional EPA 1 (Onshore office)
Int3	Project Manager at a Regional EPA Trade Office (Offshore)	Regional EPA 1 (Offshore office)
Int4	Director of Onshore Operations at a National EPA	National EPA
Int5	Coordinator of a regional EPA belonging to a network funded by the European Commission	Regional EPA 2 - European network

The sample covers both traditional EPAs and a regional EPA belonging to a network funded by the European Commission, thus providing insights into different governance and performance management models.

The selection criteria included direct responsibility for export promotion services, variation in organizational scale and scope, and availability of knowledgeable informants willing to participate.

### Data collection

Primary data were collected through semi-structured, focused interviews with key agency managers. This method allowed for a balance between consistency across interviews (through common thematic blocks) and flexibility to explore emerging topics.

The interview guide was organized into four main thematic areas, beginning with the profile of the respondent and the agency structure, exploring organizational roles, size, and governance. It then addressed success factors in export promotion, gathering perceptions of key drivers and barriers to effectiveness. Another thematic area examined the service portfolio and operational practices, specifically looking into the types of services offered and their delivery mechanisms. Finally, the guide focused on monitoring and evaluation practices, including the use of performance indicators, and challenges encountered. Sample questions from the guide included, "How do you monitor and evaluate the implementation of recommended actions by firms?" or "How do you assess whether the expected results are achieved after service delivery?"

Interviews were conducted either in person or via videoconference, depending on participant availability. Each interview lasted between 20 and 45 minutes. All interviews were audio-recorded (with participant consent) and fully transcribed for subsequent analysis.

In addition to interviews, secondary data were collected, including agency strategic plans and reports, publicly available performance evaluations, institutional websites, and policy documents. Triangulating primary and secondary sources helped validate interview findings and provided a richer understanding of each case's context.

### Data analysis

Data analysis proceeded in two main stages:

First, a descriptive coding process was applied to the interview transcripts and documents. Initial codes were organized around the main elements of the Logic Model: resources, activities, outputs, outcomes. Additional codes captured dimensions such as efficiency, service quality, and client satisfaction.

Second, an interpretative thematic analysis was conducted to identify patterns, similarities, and differences across cases. Explanation building techniques were used to develop causal narratives linking organizational practices to performance management maturity ( Yin, 2018).

Comparative cross-case analysis enabled the identification of factors facilitating or hindering the adoption of Logic Model-based PMSs, variations in the sophistication of performance monitoring practices across organizational levels, and common challenges along with innovative solutions implemented by different agencies.

To enhance the credibility and trustworthiness of the findings, a study protocol was followed consistently throughout data collection and analysis phases. Triangulation with secondary data sources and member checking with interview participants were employed. No qualitative analysis software was used as all coding and interpretation were conducted manually by the researchers.

## Findings

This section presents the results of an exploratory multiple case study involving interviews with five managers from EPAs operating at different geographic levels ( Ruiz-Coupeau, 2025): provincial (Int1), regional onshore (Int2), regional offshore (Int3), national (Int4), and regional/European (Int5). The findings are structured using the components of the Logic Model—Resources, Activities, Outputs, and Outcomes in addition to efficiency, cost-effectiveness, service quality, and customer satisfaction to assess and compare the extent to which PMSs are adopted and formalized across agencies with different mandates and operational scopes.

### Resources (Inputs)

Resources in the context of EPAs include financial support from government programs, human capital (staff, consultants, experts), operational infrastructure (e.g., overseas offices), and tools such as CRMs or databases. The level of availability and management of these resources directly influences the ability to implement structured PMS.

Int4 emphasized the role of national-level data infrastructure and institutional coordination:

*“We conduct analyses of export data segmented by company size and geographic region. This allows us to track the number of exporting firms—new, regular, or inactive—and evaluate our export base expansion.”*

Int3 illustrated the importance of decentralization and international presence as an input:

*“We’ve been operating for over 10 years in India and nearly four in Southeast Asia. Being physically present lets us gather real-time intelligence and build trust with local stakeholders and Andalusian companies.”*

Int5 described how long-term EU funding supports capacity development:

*“Programs like this run for seven years. This allows us to strengthen institutional capacity, hire skilled staff, and improve our services incrementally.”*

In contrast, Int1 signalled limited structuring in how resources are used:

“We don’t have a systematic approach to resource management. Most of our responsiveness comes from active listening and informal feedback from companies.”

### Activities

Activities refer to the core interventions of EPAs, which typically fall into four service categories ( Leonidou *et al.*, 2011): (1) information provision, (2) education and training, (3) financial support, and (4) export facilitation. The degree to which these activities are monitored and assessed varies.

Int2 offered a detailed example of structured export facilitation:

*“We design four-year country plans with annual programming for each selected sector. After each action, we prepare reports and collect satisfaction surveys to assess effectiveness and improve the next year’s planning.”*

Int3 described how export facilitation and market intelligence are delivered abroad:

*“We help firms prepare agendas for business meetings with pre-validated potential clients. We also deliver commercial prospecting reports and identify strategic partners for Andalusian companies.”*

Int1 referred to offering training and advisory services:

*“We run several programs, each with different services. These include training sessions, follow-up visits, and results surveys. We adapt our support depending on the firm’s progress.”*

However, the monitoring of activities was less systematized at the provincial and offshore levels, relying more on personal interaction and reactive feedback than formal PMS tools.

### Outputs

Outputs are the immediate results of EPA activities—such as the number of companies trained, missions organized, or advisory sessions conducted. Most agencies rely on post-activity feedback to evaluate these outputs.

Int2 reported formal tracking of outputs:

*“After each action, both the local office and headquarters submit a report. We also distribute surveys to the participating companies to evaluate the relevance and usefulness of the action.”*

Int5 linked outputs directly to performance indicators:

*“Each time a cooperation agreement is signed, or a productivity improvement is achieved, we submit an impact report to the European Commission. We ask clients to complete a 15-question survey assessing results, efficiency, employment, and innovation.”*

At the provincial level, output tracking remained more descriptive than analytical:

Int1: *“We send out satisfaction surveys and examine whether indicators like export volume or number of markets served increased, but it’s not part of a structured dashboard.”*

Int3 highlighted relational feedback mechanisms:

*“We collect satisfaction reports and maintain constant contact with companies during and after projects. Most feedback is positive, and we use it to adjust our services.”*

### Outcomes

Outcomes reflect medium- to long-term effects of EPA activities on company performance, including expanded international sales, improved competitiveness, job creation, or innovation. These were more robustly measured at the national and European levels. They are central to evaluating cost-effectiveness and broader program impact.

Int4 described outcome evaluation through public data and CRM integration:

*“We follow up with firms months after their participation to understand how their export activity evolved. Satisfaction surveys are combined with data from our CRM to assess overall impact.”*

Int5 detailed an ex-post evaluation strategy:

*“We ask firms to report the expected impact of our support—for example, an increase in sales—and one year later, we follow up to compare expectations with actual outcomes.”*

Int1 focused on firm-level outcome indicators but with limited tracking mechanisms:

*“We consider a service successful when we see increases in international turnover, new markets, or new hires in the export department. But our evaluation depends largely on what companies report informally.”*

Int3 underscored qualitative insights:

*“We rely on conversations to understand whether our services are effective. Companies appreciate ongoing support throughout the internationalization process, but we don’t track outcomes with KPIs.”*

In terms of efficiency, only Int5 referenced practices such as calculating the ratio of outcomes to resource inputs or tracking productivity over time.

### Service quality

Service quality indicators such as timeliness, thoroughness, and relevance were largely assessed through satisfaction surveys and informal feedback. While all agencies valued quality, their approaches to monitoring and enhancing it varied in structure and intensity.

To illustrate a structured approach to assessing service quality, Int2 highlighted the use of post-service questionnaires designed to capture client perceptions of utility and impact:

“ *We evaluate service quality through questionnaires—firms are asked which services were most useful and why.”*

At the offshore regional level, Int3 emphasized the importance of personalization and logistical support in enhancing the perceived quality of services delivered during international visits:

*“We care about quality a lot. For instance, we offer personal assistance to firms visiting India or Malaysia, including help with logistics, hotels, and agendas. That makes a big difference.”*

From the perspective of a European network node, Int5 described a more systemic and data-driven approach to quality management, incorporating specific dimensions and corrective actions:

*“We monitor quality dimensions like accuracy, thoroughness, and accessibility. If results are below expectations, we reallocate staff and adapt communication strategies.”*

### Customer satisfaction

Customer satisfaction was a critical but variably formalized performance dimension across agencies.

Int5 described a structured EU-mandated feedback mechanism:

*“Client satisfaction is assessed independently through surveys sent directly by the European Commission to participating firms. We use the results to adjust our services.”*

Int4 used satisfaction as a basis for program redesign:

*“All participants receive a survey after activities. We also follow up months later to evaluate satisfaction over time and adapt our instruments accordingly.”*

Int1 and Int3 used relational feedback:

Int1: *“We rely on what companies tell us during calls or visits. If they’re happy, we take it as a sign we’re doing well.”*
Int3: *“We collect qualitative feedback through conversations. It helps us see what worked and what needs to change.”*

***Comparative maturity of logic model components across the four agencies***

The European network and national-level EPAs demonstrated the highest alignment with logic model practices, particularly in integrating resources with planned activities and measuring both outputs and long-term outcomes using formal indicators and digital tools. Regional agencies showed intermediate levels of PMS maturity, with a mix of structured and ad hoc practices. The provincial agency exhibited the most informal PMS, relying heavily on relational trust and active listening rather than standardized procedures.

This comparative view reveals that the application of logic model components within EPAs correlates strongly with their territorial scope, mandate complexity, and institutional embeddedness. Agencies with broader mandates and more access to long-term funding frameworks (e.g., EU-level programs) are more likely to formalize their PMS and embed logic model thinking into strategic planning and evaluation routines.

## Development of the PMS using the logic model

The design of a PMS for EPAs requires a structured, logical, and participative approach, considering the unique complexity of export promotion activities. The Logic Model provides a conceptual and operational framework to guide this process. Its application ensures that the interventions of the agency are linked through causal chains to the intended results, fostering transparency, strategic clarity, and adaptability ( Hatry, 2006; McLaughlin & Jordan, 2015).

The Logic Model visualizes the logical relationships between inputs, activities, outputs, and the different levels of outcomes—short-term, intermediate, and long-term. Its use in EPAs as a base to design PMS serves not only for services evaluations but, crucially, for service design and operation monitoring, enabling agencies to refine their strategies and improve performance progressively ( Poister, 2015).

Based on the analysis of the case studies and the relevant literature, the development of a PMS through the Logic Model involves several critical phases that are interconnected and iterative rather than strictly linear ( Hatry, 2006; McLaughlin & Jordan, 2015).

### Stage 1: Collect the relevant information

The first phase consists of systematically collecting relevant information to construct a comprehensive understanding of the EPA's strategic orientation, operational context, and performance logic. This involves reviewing internal documentation such as strategic plans, service protocols, past evaluations, and organizational reports to identify current practices and implicit theories of change ( Hatry, 2006; McLaughlin & Jordan, 2015). Complementing this desk research, interviews and workshops with internal staff—such as program managers, evaluation officers, and frontline personnel—are essential to uncover informal routines, contextual constraints, and experiential insights that are often absent from formal records. These inputs provide the diagnostic foundation on which to structure the Logic Model's causal assumptions and performance indicators.

Equally important is the engagement of external stakeholders who influence and are affected by export assistance. EPAs operate within complex ecosystems that include domestic institutions (e.g., ministries, chambers of commerce, business councils) and international actors (e.g., the World trade organization (WTO), International Trade Centre (ITC), European Commission), all of whom contribute to shaping export policy and service delivery ( Cavusgil & Yeoh, 1994; Kahiya, 2024; Schembri *et al.*, 2019). SMEs, as primary beneficiaries, often express their needs indirectly through representative bodies, making stakeholder consultations—via interviews, surveys, or participatory workshops—vital to ensure that the PMS reflects real-world expectations and constraints ( Freixanet, 2012; Tinits & Fey, 2022). These engagements help clarify interdependencies, information flows, and service gaps, ultimately producing a baseline assessment that anchors the Logic Model in both institutional realities and stakeholder-driven priorities ( Poister, 2015).

### Stage 2: Define the problem and its context

The second phase involves defining the problem which requires identifying the mismatch between the support SMEs need and the way assistance is currently structured. Export assistance should be grounded in a clear understanding of firm-level constraints—such as informational gaps, procedural hurdles, or limited financing—which often reflect broader systemic challenges ( Crick & Czinkota, 1995; Freixanet, 2012). The core issue lies not only in what interventions are needed, but also in determining which firms should be prioritized. Some advocate for supporting the most vulnerable firms with limited resources, while others argue for targeting those with high growth potential ( Francis & Collins-Dodd, 2004; Miocevic, 2013). Operationalizing effective assistance thus requires EPAs to guide firms along a structured export journey while adapting interventions to specific needs and conditions ( Kahiya, 2024).

Firm segmentation plays a central role in this targeting process. Traditional approaches classify firms by their stage of internationalization, with early-stage exporters benefiting from awareness and training, and experienced ones requiring financial or mobility support ( Crick & Czinkota, 1995; Welch & Wiedersheim-Paul, 1979). Yet, this model struggles to reflect the realities of accelerated and non-linear internationalization paths, especially among high-growth or tech-based ventures ( Belhoste *et al.*, 2019; Oviatt & McDougall, 1994). Alternative segmentation strategies based on business models, managerial capabilities, or export destinations offer more flexible and realistic criteria for tailoring support ( Fischer & Reuber, 2003). EPAs must therefore balance structured targeting with adaptive design, ensuring their support mechanisms match both firm diversity and shifting market dynamics.

Contextual factors further shape how problems are defined and addressed. National economic maturity, regulatory quality, and global integration levels influence both the demand for and impact of export assistance ( Freixanet & Churakova, 2018; Ribeiro *et al.*, 2020). In emerging economies, EPAs often lead innovation in support services, leveraging supranational guidance and funding from bodies like the WTO and ITC ( Kahiya, 2024). Meanwhile, political shifts, fiscal constraints, and administrative capacity also affect how EPAs are evaluated—either by operational performance or strategic engagement ( Lederman *et al.*, 2010; Schembri *et al.*, 2019). Defining the problem therefore demands a multi-level perspective that considers both firm-specific barriers and the institutional context in which EPAs operate.

### Stage 3: Define the elements of the Logic Model

Once the performance context and stakeholder needs have been clarified, the next step in developing a Logic Model-based PMS for EPAs is to define the elements of the model itself. These elements include inputs, activities, outputs and outcomes, each representing a link in the causal chain from resource allocation to societal benefit. A well-defined Logic Model articulates how agency interventions are expected to lead to desired changes, providing a framework for monitoring, learning, and adaptation ( Hatry, 2006; McLaughlin & Jordan, 2015).

Inputs refer to the tangible and intangible resources mobilized by EPAs to deliver export assistance. These include financial allocations from government and EU programs, human capital such as EPA staff and advisors, physical infrastructure (e.g., trade offices and embassies), and digital systems for client management and service tracking. Also included are the operational budgets for trade missions, training programs, export grants, and advisory services ( Leonidou *et al.*, 2011). These inputs serve as the foundation for implementing export support programs and must be inventoried and tracked to ensure resource efficiency.

Activities, or interventions, are grouped into four main categories as identified in the literature ( Kahiya, 2024; Leonidou *et al.*, 2011). The information-related activities focus on disseminating knowledge through market intelligence, export directories, and guidance on procedures. Education and training-related interventions include seminars, technical workshops, e-commerce training, and export counselling, often delivered by EPAs’ advisors or subcontracted experts. Financing-related initiatives provide firms with improved access to working capital, export credit insurance, and export grants. Lastly, trade mobility-related activities facilitate firm participation in trade fairs, business missions, and matchmaking services, often through foreign trade offices or embassies. This categorization captures both the onshore support by EPAs and the offshore facilitation by trade representatives abroad.

Outputs are the immediate, measurable results of these activities. These include the number of firms trained, services delivered, clients supported, and events organized. For example, outputs might reflect how many SMEs received technical training, the number of firms participating in a trade mission, or the total value of financial support granted. While not traditionally part of the Logic Model, client satisfaction surveys are incorporated at this stage to assess perceived service quality and alignment with expectations ( Freixanet, 2022).

Outcomes are categorized along a time horizon. Short-term outcomes reflect immediate organizational changes, such as establishing an export department, launching international strategies, receiving export subsidies, or forming distribution agreements abroad ( Filipe Lages & Montgomery, 2005). Intermediate outcomes relate to firm performance metrics such as export volume, sales growth, profitability, and export intensity ( Cavusgil & Yeoh, 1994). These reflect how the intervention has shifted behavior and capabilities within firms. Long-term outcomes, meanwhile, aim to reduce domestic market dependence, stabilize demand cycles, and increase the resilience and international competitiveness of SMEs. These changes contribute to sustained export activity and reduced vulnerability to external shocks.

Additionally, we will add to the Logic Model the impact, referring to macro-level effects such as export growth, market diversification, foreign exchange accumulation, and employment creation ( Broocks & van Biesebroeck, 2017; Martincus & Carballo, 2010). These long-term societal benefits validate the public investment in export promotion and are typically assessed through national statistics and longitudinal studies. EPAs thereby play a key role in enhancing the global integration and economic dynamism of the countries they serve.

### Stage 4: Draw the logic model

Stage 4 involves translating the conceptual structure of the export assistance program into a visual Logic Model that clearly links inputs, activities, outputs, and outcomes. This visual representation enables EPAs to articulate how their resources and interventions are expected to produce measurable changes at the firm and national levels. Drawing on the typology outlined by Leonidou *et al.* (2011) and Kahiya (2024), the model organizes interventions into four core categories—information, education and training, financing, and trade mobility—and traces their progression from service delivery to short-term organizational changes, intermediate performance improvements, and long-term resilience and competitiveness.

The Logic Model also establishes a results chain that supports performance monitoring and strategic decision-making. Inputs such as staff, consultants, and financial resources lead to structured activities like training sessions, trade missions, and funding schemes. These, in turn, generate outputs such as the number of firms assisted or training hours delivered. Outcomes are grouped by temporal proximity—short-term effects such as export strategy formulation, intermediate outcomes like sales growth, and long-term changes such as market diversification. At the macro level, impacts reflect broader societal benefits, including export growth and job creation, offering a comprehensive and communicable framework for assessing EPA effectiveness.

### Stage 5: Verify the Logic Model with stakeholders

Once the draft Logic Model is built, the fifth phase entails its validation. Validation should be participative, involving managers, field officers, SMEs representatives, and policy makers, ensuring that the causal logic is realistic, and the indicators are meaningful and feasible to monitor. Validation workshops allow for the adjustment of the model based on operational realities and user feedback, reinforcing the idea that the PMS must be a living tool that evolves with experience and environmental changes ( Radnor & Barnes, 2007).

**Challenges when designing PMS in EPAs:**

Throughout the process, the Logic Model must be adapted to the specificities of EPAs. These organizations deal with particular challenges that must be addressed in the design of their PMS:

First, outcomes in export promotion are often the result of cumulative and multi-causal processes, where the agency's intervention is one among many contributing factors. Therefore, the attribution problem is central. Agencies must design indicators that capture contributions rather than attributing direct causality, using techniques such as contribution analysis or tracing intermediate changes ( Rose, 2007).

Second, the time lag between the delivery of services and the realization of outcomes can be considerable. Export promotion impacts often materialize years after initial support. PMSs must be designed to track changes over time, using longitudinal surveys or client follow-ups at different intervals, as suggested by Freixanet and Churakova (2018).

Third, the diversity of services offered, and the heterogeneity of client firms demand flexible and differentiated performance indicators. Not all SMEs have the same export potential or require the same type of support. Performance systems should allow for segmentation by firm profile, service type, and export maturity level ( Seringhaus & Rosson, 2012).

Fourth, measuring service quality and client satisfaction is essential, not only for accountability but also for continuous improvement. Quality dimensions such as relevance, timeliness, customization, and client perceived value should be systematically integrated into the PMS through structured satisfaction surveys and relational feedback mechanisms ( McLaughlin & Jordan, 2015).

Finally, efficiency and cost-effectiveness indicators should be incorporated to monitor the optimal use of resources. While effectiveness measures the extent to which objectives are achieved, efficiency assesses how economically the agency achieves its results, a dimension increasingly demanded by funding authorities and taxpayers ( Pollitt & Bouckaert, 2011).

In sum, developing a PMS through the Logic Model requires a combination of strategic clarity, participative design, flexibility to adapt to different client trajectories, and a robust system of monitoring and evaluation that captures both quantitative and qualitative dimensions of performance. By systematically applying these principles, EPAs can enhance their capacity to demonstrate impact, learn from experience, and contribute more effectively to the internationalization of SMEs and economic development goals.

## Extended illustrative case: the services of an EPA belonging to a European network

The EPA belonging to a European network constitutes a paradigmatic case of how a Logic Model-based PMS can be effectively implemented in an EPA. Coordinated by the European Commission, the network represents the world’s largest support network for SMEs with international ambitions, operating through more than 600 member organizations across over 60 countries. The case illustrates the operationalization of the Logic Model principles into an EPA belonging to a complex, multi-level, and multi-service international network.

The network operates under long-term financial frameworks, such as the COSME programme and, more recently, the Single Market Programme. This funding structure provides network members with the stability required to implement strategic planning cycles spanning several years, a crucial factor for embedding a Logic Model-based PMS within its operations.

**Figure 2.**
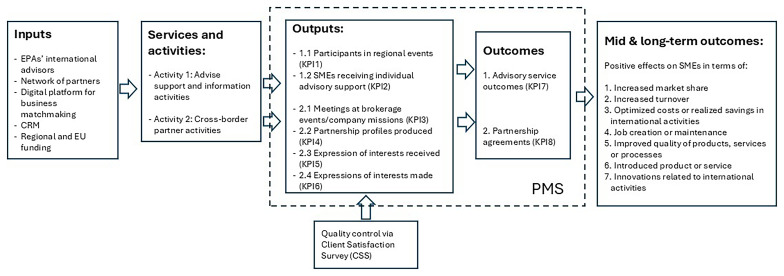
A proposed logic model of the services provided by the EPA.

The Logic Model of the services and activities is shown in the following figure ( Figure 2):

The resulting Logic Model-based PMS in which efficiency ratios where added is shown in Table 2:

**Table 2.** Logic Model-based PMS.

Data item	Description
**Outputs**	
KPI1	Participants in regional events
KPI2	SMEs receiving individual advisory support
KPI3	Meetings at brokerage events/company missions
KPI4	Partnership profiles produced
KPI5	Expression of interests received
KPI6	Expressions of interests made
**Outcomes**	
KP7	Advisory service outcomes
KP8	Partnership agreements
**Efficiency ratios**	
Ratio 1 (ASO/FTE)	Number of advisory service outcomes (KPI7) over the total number of FTEs ^2^
Ratio 2 (PA/FTE)	Number of partnership agreements (KPI8) over the total number of FTEs

Figure 1 and Table 2 were developed by the authors through the integration of interview findings and secondary data, including the structured performance management system of the European network, within the logic model framework. The referenced system is standardized across the network, which does not permit the use of context-specific indicators at the local node level.

The Inputs mobilized by the network are considerable and varied. Financially, the network relies on European Commission grants co-financed by participating organizations. Human resources comprise highly qualified professionals from the EPA, including international advisors. Technological inputs include centralized Client Relationship Management (CRM) systems and digital platforms for business matchmaking and service monitoring. Relational inputs are provided through the formalized partnerships between the organizations forming the network. The EPAs has thus multi-input architecture necessary to deliver complex export promotion interventions.

The activities conducted are structured into modular service packages, carefully aligned with the strategic objectives set by the European Commission. These include advisory support and information services, such as providing guidance on EU legislation, access to finance and funding opportunities, and organizing local or regional events. Additionally, cross-border partnership activities are carried out through brokerage events, trade missions, and partner search services, which assist in drafting international partnership profiles and managing expressions of interest both received and made. Each activity is standardized across the network while remaining adaptable to regional contexts and EPAs, ensuring coherence and flexibility in service delivery.

A distinctive feature of the service delivery model is the concept of the *Client Journey*, particularly for firms receiving personalized services. The process begins with an initial diagnostic phase, during which client needs, capabilities, and objectives are systematically assessed. This assessment leads to the formulation of an action plan, a structured document that outlines the services to be delivered, the expected outputs, and the anticipated short- and intermediate-term outcomes. The action plan thus serves as a practical operationalization of the Logic Model within individual client engagements, providing a roadmap for service provision and a baseline for subsequent evaluation.

Outputs are rigorously monitored through standardized reporting procedures in a platform. Key performance indicators include the number of participants in regional events organized by the EPA (KPI1), the number of SMEs supported with specialized EPA services (KPI2), the number of meeting organised during matchmaking events and trade missions (KPI3) and the number of partnership profiles produced (KPI4) and expression of interest received (KPI5) and made (KP6) to these partnership profiles. This systematic output monitoring ensures comparability across regions and consistency with the performance indicators mandated by the European Commission.

Importantly, the network does not limit its PMS to tracking outputs but places significant emphasis on measuring Outcomes. Short-term outcomes include advisory service outcomes (KPI7) such as improvements in client firms' knowledge about international markets, regulatory compliance, successful acquisition of EU funding, and the formation of partnership agreements (KPI8) like international distribution agreements. Intermediate and long-term outcomes, though more difficult to capture, are assessed through client follow-up surveys conducted 12 months after service delivery, which examine the positive effects on SMEs in terms of increased market share, increased turnover, optimized costs or realized savings in international activities, job creation or maintenance, improved quality of products, services or processes, the introduction of new products or services, and innovations related to international activities.

The PMS also integrates efficiency measures by calculating outcome-per-resource ratios (Ratios 1 and 2). These FTE-based efficiency ratios are not merely formalities; they are actively used to inform resource allocation and guide strategic adjustments, consistent with the principles of evidence-based management advocated by Hatry (2006) and Pollitt and Bouckaert (2011). Nodes consistently demonstrating low efficiency performance may be subject to corrective actions, reinforcing the system’s emphasis on accountability.

Another critical dimension of the PMS is the emphasis on Service Quality. Client satisfaction is systematically measured through standardized surveys (called Client Satifaction Surveys – CSS) assessing relevance, timeliness, professionalism, and perceived value of services. The results are aggregated and analyzed at both the local node and European levels, with client feedback serving as a key input for continuous service improvement.

The Logic Model-based PMS thus stands out not only for its comprehensiveness but also for its dynamic nature. The system is designed to adapt based on ongoing monitoring data, evaluation findings, and changes in the external environment. The integration of short-term feedback loops (e.g., immediate client satisfaction surveys) and longer-term impact assessments enables the network to practice adaptive management, constantly refining its services and strategies.

However, the successful implementation of such a sophisticated PMS is contingent upon several enabling factors. These include the existence of stable and substantial financial resources, a highly professionalized workforce, advanced digital infrastructures, and a strong culture of evaluation and learning. As Freixanet and Churakova (2018) noted, without these structural conditions, the replication of the network model in other contexts may face significant limitations.

Nevertheless, the EPA belonging to a European network provides a compelling example of how a Logic Model-based PMS can significantly enhance the strategic coherence, operational efficiency, and outcome orientation of an EPA. It demonstrates that with the right institutional design, resources, and cultural commitment, EPAs can move beyond mere output counting toward genuine impact assessment and strategic learning, contributing more effectively to SME internationalization and, ultimately, to broader economic development objectives.

## Discussion and future research directions

The results of this research support the value of the Logic Model as a structuring and operational tool for designing and implementing PMSs in EPAs. By explicitly mapping the relationships between resources, activities, outputs, and outcomes, the Logic Model offers a framework capable of enhancing strategic clarity, accountability, and continuous learning within public organizations ( Hatry, 2006; McLaughlin & Jordan, 2015).

However, the case studies reveal that the successful application of the Logic Model depends heavily on institutional factors such as organizational scale, resource availability, external accountability requirements, and the internal culture of evaluation and learning ( Pollitt & Bouckaert, 2011). Larger agencies, such as national and European-level EPAs, which operate under strong external accountability mechanisms and benefit from stable funding, are better positioned to develop sophisticated PMSs. In contrast, regional and provincial agencies, often working in resource-constrained environments, tend to focus more on immediate service delivery than on systematic performance monitoring.

These findings align with Poister’s (2015) argument that performance management is not only a technical exercise but also a political and organizational one. The establishment of PMSs is influenced by the incentives, resources, and strategic priorities prevailing within each organization.

One of the most persistent challenges identified is the difficulty of measuring outcomes in the field of export promotion. The export behavior of firms is influenced by a myriad of internal and external factors, making direct attribution of success to EPA interventions problematic ( Freixanet & Churakova, 2018; Rose, 2007). Moreover, many of the effects produced by export support services—such as enhanced knowledge, strategic reorientation, or network expansion—are intangible and occur over long time horizons ( Freixanet, 2012).

The regional EPA belonging to a network case illustrates both the potential and the limitations of Logic Model-based PMSs when applied to complex business support networks. The network’s node structured client journeys, personalized action plans, and multi-level monitoring practices represent best practices in aligning service delivery with strategic objectives. However, even within the network, challenges persist in standardizing performance indicators across diverse contexts, capturing soft outcomes, and attributing results in multi-causal environments.

In light of these challenges, this study emphasizes the urgent need for further research focused specifically on PMSs in business support networks. As many researchers noted ( Costumato, 2021; Herranz, 2010; Radnor & Barnes, 2007), the application of performance management in networked environments introduces additional complexities compared to traditional hierarchical organizations. Coordination among diverse actors, harmonization of services, and collective accountability mechanisms require new models of PMS design and implementation.

This study also highlights the potential of benchmarking as a mechanism to support agencies in progressing toward more structured, Logic Model-based PMSs. The differences observed, from informal, feedback-driven approaches to institutionalized performance systems, suggest that sharing practices across agencies can foster convergence. As shown in the literature, benchmarking enhances learning and promotes standardization in public sector performance frameworks ( Arnaboldi *et al.*, 2015; Bovaird & Loeffler, 2023; Triantafillou, 2007).

Future research should address several priority areas. First, more work is needed on how to adapt Logic Models to multi-actor governance ( Costumato, 2021; Herranz, 2010). Research could investigate methods for developing shared theories of change across network partners, ensuring alignment while respecting organizational diversity ( Pollitt & Bouckaert, 2011).

Second, there is a critical need to develop and validate indicators that can capture soft, relational, and knowledge-based outcomes, which are often the first observable effects of export promotion services ( Freixanet, 2012; Freixanet & Churakova, 2018). In addition, future studies could expand data collection to build a comparative table of KPIs, ratios, and indicators. This would make differences in PMS formality and orientation clearer, revealing how agencies vary between basic activity tracking and more advanced outcome or efficiency measures, and clarifying the role of the Logic Model in shaping performance management maturity.

Third, longitudinal studies should be conducted to better understand the temporal dynamics of export promotion impacts. Tracking firm behavior and performance over extended periods would provide richer insights into the causal linkages between agency support and export outcomes ( Rose, 2007; Tinits & Fey, 2022).

Fourth, future studies should explore the integration of digital technologies into PMS design and operation. The use of advanced CRM systems, big data analytics, and artificial intelligence tools could greatly improve the capacity of EPAs and networks to monitor, predict, and adapt their interventions in real-time ( Siyu, 2025).

Finally, current EPA performance systems remain primarily focused on economic indicators such as export growth and competitiveness, while social and environmental dimensions are rarely integrated in a structured manner. This narrow scope represents a limitation, especially as sustainability has become a central policy priority in the EU and internationally. The Logic Model, however, offers the flexibility to incorporate multidimensional indicators, such as environmental impact, green exports, or inclusive job creation, into future PMS design. Research shows that sustainability measures remain among the least applied in public sector performance systems ( Adams *et al.*, 2014), though recent literature on public sector sustainability management controls ( Bauer & Greiling, 2025) confirms that public organizations are adopting controls aligned with Environmental, Social y Governance (ESG) and Sustainable Development Goals (SDGs), the implementation is often incomplete, with limited social dimension integration and lack of holistic design.

## Conclusion

This study has proposed the application of the Logic Model as a structuring and operational framework for the development of PMSs in EPAs. Using an exploratory multiple-case study, including an extended analysis of a regional EPA belonging to a European network, the research has provided insights into two guiding research questions.

### First, how are PMSs designed in EPAs?

The findings show significant variation in sophistication and formality across agencies. National and European-level EPAs, operating under strong accountability requirements and with access to stable resources, have advanced in structuring their PMSs around the Logic Model principles ( Hatry, 2006; McLaughlin & Jordan, 2015; Poister, 2015). In contrast, regional and provincial agencies often rely on output-focused measurement systems along with informal practices. Nonetheless, the Logic Model proved to be a coherent and flexible methodology for linking resources, activities, and outcomes, while also encouraging strategic learning and stakeholder engagement ( Coryn *et al.*, 2010).

### Second, how can PMSs be particularised to the needs and realities of EPAs using the Logic Model framework?

The case studies demonstrate that successful PMS implementation depends on a set of enabling conditions. These include: (i) stable and long-term funding frameworks, (ii) investment in data infrastructure and digital tools, (iii) sufficient internal capacity and professionalized staff, and (iv) an organizational culture that values evaluation and continuous learning ( Hatry, 2006; McLaughlin & Jordan, 2015; Poister, 2015). Without these conditions, attempts to replicate advanced PMS models are likely to face serious limitations.

The regional agency of the European network case provides a concrete illustration of these dynamics. Its Logic Model-based PMS integrates efficiency ratios, outcome indicators, and client satisfaction data into strategic decision-making. For example, efficiency indicators such as the ratio of advisory service outcomes per staff member is systematically used to reallocate resources across network nodes. When a node shows persistently weak effectiveness, corrective measures, including staff reallocation or redesign of service portfolios, are adopted. This process reduces the risk of “decoupling” ensuring that performance indicators genuinely inform managerial choices rather than being treated as symbolic compliance ( Jabbouri *et al.*, 2022). Moreover, the integration of client feedback has led to the redesign of services, aligning them more closely with SME needs and reinforcing the adaptive capacity of the agency.

In sum, by integrating the Logic Model into their performance management practices, EPAs can move beyond mere output tracking toward genuine impact assessment, learning, and strategic adaptation. While challenges remain, particularly regarding outcome attribution, measurement of intermediate results, and longtime lags ( Freixanet, 2022; Rose, 2007) the framework helps agencies strengthen accountability and enhance their contribution to SME internationalization and economic development. Future research should further explore how Logic Models can be adapted to multi-actor governance and digitalized environments, where the complexity of export promotion support is most acute Costumato, 2021; Herranz, 2010; Radnor & Barnes, 2007)

## Ethics and consent

This research adopts a positivist approach and relies on exploratory and descriptive analysis through qualitative interviews with export promotion agency managers. The primary aim of the study is theoretical contribution, not practical application. Interviewees are not classified or analyzed based on gender, race, economic status, or any personal characteristic. Personal attributes, including psychological or social traits, are not considered at any point. Managers speak solely about their roles within their respective organizations and the organizational context in which they operate. No personal, psychological, or social data are collected, and individuals do not make decisions, solve problems, or participate in any form of experimental manipulation. The data collected are purely descriptive and exploratory in nature, and all information is processed anonymously and in aggregate form. At the beginning of each interview, managers were informed that all information would be treated completely anonymously and in aggregate. They were assured that the data collected would be used solely for research purposes and never disseminated outside the academic field. It was explicitly stated that neither their names, nor the names of their companies, customers, or suppliers would be disclosed at any stage. Given that consent was explicitly addressed during the interview and captured in the audio recording, verbal consent was deemed sufficient, and no additional written consent was required. Due to the nature of the study, no ethics committee approval was required by the funding body or the host institution.

## Figures and Tables

**Annex 1.** 

Cases	Short description	Information services	Training services	Financing services	Trade mobility services
**Provincial chamber of commerce**	The Chamber functions as a public law corporation and plays a pivotal role in supporting the regional economic development of the province. As an institutional actor with a mandate to represent, promote, and defend the general interests of commerce, industry, and navigation, the Chamber operates at the intersection of public policy and private enterprise, offering a broad array of services to businesses, including a specific focus on small and medium-sized enterprises (SMEs). Its activities span multiple domains, each aligned with its overarching mission to foster entrepreneurship, competitiveness, and internationalization.	The Chamber acts as a knowledge intermediary by gathering, processing, and disseminating relevant economic, legal, and sectoral data. This includes the publication of market intelligence reports, statistical data on trade flows, regulatory updates, and economic forecasts. These informational outputs are disseminated through various channels, such as digital newsletters, dedicated sections on the Chamber's website, and thematic seminars. By doing so, the institution supports evidence-informed decision-making and reduces information asymmetries for businesses operating within a complex and evolving economic environment.	The Chamber provides structured programs aimed at enhancing managerial and operational competencies across various sectors. These include vocational training, professional certification programs, and executive development initiatives tailored to both early-stage entrepreneurs and established business leaders. The Chamber collaborates with educational institutions and leverages its own training infrastructure to deliver these services either in-person or via online platforms. Through these programs, it not only promotes lifelong learning but also addresses skill gaps critical to the regional labor market.	The Chamber plays an active role in supporting local businesses through financial services. It awards direct subsidies funded by the European Union aimed at promoting innovation, digitalization, and internationalization projects. Furthermore, the Chamber offers advisory services to help enterprises identify additional sources of public grants and subsidies. It also collaborates closely with financial entities and governmental agencies to facilitate access to a broader range of financial resources.	The Chamber undertakes a comprehensive set of initiatives aimed at promoting international trade. These include organizing trade missions, coordinating participation in international fairs, and offering tailored consultancy services on market entry strategies. They use the offshore network of official Spanish of chamber abroad. The Chamber also provides legal and administrative support for export documentation and compliance, including the issuance of certificates of origin and other trade-related formalities. Furthermore, it maintains active cooperation with Spain’s national network of chambers and international business promotion agencies, thereby serving as a critical node in the internationalization ecosystem for firms in the region.
**Regional EPA**	The agency is a public company owned by the Regional Government, dedicated to promoting companies and products from the region in international markets. Its primary objectives include increasing regional investment abroad, enhancing the positioning of regional companies internationally, and expanding the number of businesses with a global presence.	The agency provides market intelligence and advisory support that are vital to informed decision-making. Companies have access to detailed country and sector reports, trade statistics, and risk analyses. The agency also issues regular alerts about international tenders and commercial opportunities, and maintains a knowledge repository covering trade regulations, certification requirements, customs processes, and logistical considerations. In addition, customized market research services are available for companies with specific information needs, enabling them to gain targeted insights that can shape their export strategies.	The agency delivers structured programs to build the international business competencies of local firms. This includes organizing workshops, seminars, and training sessions that cover essential topics such as global marketing strategies, international logistics, digital tools for export, and legal compliance in foreign markets. A dedicated internationalization training school offers specialized curricula designed for professionals seeking to enhance their skills in global trade. These training opportunities are often tailored to meet the specific needs of priority sectors or target markets, aiming to equip businesses with the internal capabilities necessary for successful international engagement.	The agency subsidizes the participation of firms in trade fairs, international promotional events, and commercial missions, thereby reducing the financial burden associated with these activities. It also facilitates access to subsidized consultancy services focused on export planning and foreign market entry. Moreover, the agency serves as an intermediary in connecting companies with broader financial instruments available at the regional and European levels, helping them navigate complex funding landscapes and secure necessary resources for global expansion.	Export facilitating activities represent a core component of the agency’s work, focusing on the creation of commercial linkages and visibility in foreign markets. These include the coordination of both outbound and inbound trade missions that connect local businesses with potential international clients and partners. The agency organizes and supports participation in prominent trade fairs and hosts B2B matchmaking and networking events. Through a network of international offices located in multiple countries, it offers localized assistance to firms seeking to establish or grow their presence abroad. Additionally, the agency leads targeted promotional campaigns that highlight regional products and sectors in strategically selected markets. These activities are aimed at reducing transaction costs, opening new market opportunities, and fostering sustainable export relationships.
**National EPA**	The national export promotion agency operates under the authority of the Ministry of Industry, Trade and Tourism and is structured as a public business entity. Its governance framework includes a Board of Directors composed of representatives from various ministries, national business organizations, and experts in international trade.	The agency functions as a knowledge intermediary by compiling and disseminating economic data, market intelligence, and sectoral analysis. Firms can access country-specific reports, trade flows, and macroeconomic indicators, as well as receive tailored advice for specific markets. The provision of such services is intended to reduce information asymmetries and transaction costs, enabling firms to make more informed strategic decisions.	The agency provides structured programs intended to build the capacity of firms and individuals to operate in international contexts. These programs include thematic seminars, sector-specific workshops, and broader professional training courses. A notable component is a scholarship and internship scheme that combines academic training with practical experience abroad. This model is designed to address human capital deficits in international trade functions, particularly within SMEs.	The agency’s financial support mechanisms are targeted at reducing the initial costs associated with internationalization. While it does not function as a financial institution, it manages public resources that are allocated to co-finance activities such as promotional campaigns, market research, and the establishment of foreign subsidiaries. These EU funded instruments are especially relevant for SMEs, which typically face greater constraints in mobilizing resources for international expansion.	Export facilitation represents the most outward-facing dimension of the agency’s activities. It organizes trade missions, business matchmaking events, and coordinates national participation in international trade fairs. Furthermore, the agency operates a network of economic and commercial offices abroad, which provide in-market support to domestic firms. These offices play a dual role by collecting market intelligence and serving as platforms for diplomatic-commercial engagement. This infrastructure supports firms’ access to foreign markets and contributes to the implementation of national trade promotion strategies.
**Regional node of a European network of EPAs**	The agency is a regional node within a pan-European network of agencies dedicated to supporting the internationalisation of SMEs. Functioning under the auspices of the European Commission, the network operates through a distributed structure, wherein regional partners collaborate to deliver a harmonised suite of services. These partners, while embedded within specific territorial contexts, act collectively to implement the strategic objectives of the network, ensuring the alignment of local SME support mechanisms with broader EU policy goals. This node-based structure allows for a nuanced understanding of regional economic dynamics while ensuring access to the extensive resources and policy instruments of the European Union.	The network offers extensive information services that serve as a vital conduit between European policy frameworks and regional economic actors. Through personalized advisory support, online databases, newsletters, and digital platforms, the network disseminates critical information regarding EU legislation, market access conditions, standards and certifications, and funding opportunities. This role is particularly crucial for SMEs lacking internal capacity to navigate the complexity of international regulatory environments, enabling informed decision-making grounded in up-to-date intelligence.	The network provides capacity-building interventions aimed at enhancing SMEs’ strategic capabilities. This includes workshops, seminars, and tailored coaching sessions that focus on innovation management, intellectual property rights, regulatory compliance, and cross-border business strategies. These activities are designed not only to increase managerial competencies but also to foster a culture of continuous learning and adaptation among SMEs engaging with global markets.	Although the network does not directly provide financial support, it plays a facilitative role by guiding SMEs toward appropriate financial instruments and funding schemes available at both the European and national levels. Advisors within the network help identify suitable funding calls under programmes such as Horizon Europe, COSME, and regional development funds. Furthermore, the network assists in the preparation of proposals and business plans, thereby enhancing firms’ absorptive capacity for external financial resources.	The network actively engages in export facilitating activities, which constitute a core component of its mission. The network promotes international business cooperation through brokerage events, trade missions, and the establishment of transnational partnerships. Its database of business opportunities allows SMEs to search for commercial profiles. Moreover, the network advisors offer bespoke support in developing internationalization strategies, identifying target markets, and navigating logistical and legal barriers to trade. By leveraging its embeddedness in both local ecosystems and EU-wide institutional frameworks, the network enables SMEs to expand their global reach in a structured and sustainable manner.

## Data Availability

This research contains the following underlying data: Interviews with five managers from EPAs operating at different geographic levels ( https://doi.org/10.5281/zenodo.15614565) This project contains the following extended data: Semi-structured interview ( https://doi.org/10.5281/zenodo.16145365) Data are available under the terms of the Creative Commons Attribution 4.0 International license (CC-BY 4.0)

## Footnotes

^1^ We conducted interviews in Spain, a European country composed of multiple regions, each of which is further subdivided into provinces

^2^ FTE: Full Time Equivalent
