# Supplementary material for: Logic model-based performance management systems for export promotion agencies
Source: Open Res Eur. 2025 Sep 23;5:212. Originally published 2025 Aug 1. [Version 2] doi: 10.12688/openreseurope.20545.2 (PMC12498084; doi:10.12688/openreseurope.20545.2)
Supplement: Peer review history

# Reviewer report: Reviewer response for version 2

**DOI:** 10.21956/openreseurope.23123.r60994

**Published:** 2025-10-07

**Authors:** Beatriz García Cornejo, José Antonio Pérez Méndez

**Affiliations:** 1 Accounting, Universidad de Oviedo Facultad de Economia y Empresa (Ringgold ID: 83181), Oviedo, Asturias, Spain

The authors have carefully addressed all major suggestions raised in the review. The revised version now includes a clearer definition and visual representation of the Logic Model, expanded context on Spanish EPAs, an explicit discussion of multi-actor accountability and the risk of decoupling, and clarifications on the methodological procedures. In my view, one of the most valuable improvements is the rewritten conclusion, which now directly answers the research questions and illustrates the practical use of indicators through the European network case. This significantly strengthens the paper’s contribution. The discussion has also been enriched with reflections on benchmarking and sustainability, while the issue of efficiency ratios has been resolved by focusing on robust FTE-based indicators. Although the comparative table of KPIs was not included, the authors acknowledge its value and propose it as a direction for future research. Overall, the manuscript has improved in clarity, analytical depth, and transparency. I consider it suitable for acceptance in its current form.

If applicable, is the statistical analysis and its interpretation appropriate?

Not applicable

Is the study design appropriate and is the work technically sound?

Yes

Is the work clearly and accurately presented and does it engage with the current literature?

Partly

Are the conclusions drawn adequately supported by the results?

Partly

Are sufficient details of methods and analysis provided to allow replication by others?

Partly

Are all the source data and materials underlying the results available?

Yes

Reviewer Expertise:

Management accounting, management and control systems in health care, financial and efficiency analysis, agricultural economics

We confirm that we have read this submission and believe that we have an appropriate level of expertise to confirm that it is of an acceptable scientific standard.

# Reviewer report: Reviewer response for version 2

**DOI:** 10.21956/openreseurope.23123.r61328

**Published:** 2025-10-06

**Authors:** Jose M Sanchez

**Affiliations:** 1 Universidad de Cádiz, Puerto Real, Spain

The manuscript presents relevant and timely research, addressing a significant issue in the management of Export Promotion Agencies: the implementation of effective Performance Management Systems. The study addresses a topic of great practical and theoretical importance that has been scarcely explored in the academic literature. The proposal to use the Logic Model as a strategic tool is well-founded. The study is well structured, the methodology is appropriate for the exploratory objectives, and the results are presented clearly.

 The abstract is clear, concise, and complete. It presents the background, methodology, key results, and main conclusions of the study.

 The introduction effectively establishes the context and relevance of the problem. The authors clearly identify the crucial role of Export Promotion Agencies, as well as the challenges inherent in measuring their performance. The argument regarding the use of the Logic Model in this specific context is convincing. The research questions are well formulated and logically guide the development of the study.

 The qualitative multiple case study methodology is well designed and executed, providing rich and contextualised findings. The choice of a multiple, qualitative, exploratory case study is entirely appropriate for the nature of the research. It enables a deep understanding of organisational practices and challenges in their real context. The selection of cases is a strong point, as it offers a rich and diverse perspective on the phenomenon. The description of the data collection and analysis process is transparent and systematic, which reinforces the credibility of the findings.

 The presentation of the results, structured around the components of the Logical Model is logical and easy to follow. The comparative analysis between the different agencies is insightful, highlighting the variability. The discussion section effectively interprets the results, connecting them to the existing literature on performance management in the public sector. The discussion of implications for future research is valuable and opens up new avenues of study. The article represents a valuable contribution to both academic literature and professional practice in the field of export promotion. Its publication is recommended, subject to consideration of some minor revisions to strengthen its impact further.

- Although some limitations of the Logic Model are mentioned, further exploration could be made of how these limitations manifested in the cases studied and what strategies the agencies could implement to mitigate them.
- A brief discussion of how the Spanish institutional context might influence the findings (compared to other political or economic systems) would enhance the transferability of the results.
- Incorporating the perspective of beneficiary SMEs into future research could provide a comprehensive 360° view of the effectiveness of these performance management systems.

In summary, this is a solid and well-executed research that deserves to be widely disseminated. My congratulations to the authors for their excellent contribution.

If applicable, is the statistical analysis and its interpretation appropriate?

Yes

Is the study design appropriate and is the work technically sound?

Yes

Is the work clearly and accurately presented and does it engage with the current literature?

Yes

Are the conclusions drawn adequately supported by the results?

Yes

Are sufficient details of methods and analysis provided to allow replication by others?

Yes

Are all the source data and materials underlying the results available?

Yes

Reviewer Expertise:

Entrepreneurship, trust, control systems, inteorganizational relations

I confirm that I have read this submission and believe that I have an appropriate level of expertise to confirm that it is of an acceptable scientific standard.

# Reviewer report: Reviewer response for version 1

**DOI:** 10.21956/openreseurope.22230.r57606

**Published:** 2025-08-29

**Authors:** Wiro Oktavius Ginting

**Affiliations:** 1 Public Administration, University of Sumatera Utara, Medan, North Sumatra, Indonesia

An overview of the article:

 The Logic Model can be used to design and execute performance management systems (PMSs) in export promotion agencies, as outlined in the article "Logic model-based performance management systems for export promotion agencies." The article demonstrates how the Logic Model enhances accountability, transparency, and adaptive management while drawing on several case studies of Spanish EPAs, including a longer example of a European networked agency. In addition to offering useful advice for policymakers, the paper adds to the scholarly discussion on performance management.

 Evaluation:

 The manuscript is technically sound, logically organized, and well written. The methodology is sufficiently described to permit replication, and the study design, a qualitative multiple-case study, is suitable. The open availability of data and associated documentation increases transparency. The findings are supported by the conclusions, which also provide practitioners and academics with insightful information. 

 Opportunities for Development:

 There is room to improve the level of interaction with the most recent literature. Although the study uses pertinent sources, its contribution would be strengthened by including research on export promotion and public sector performance management conducted in the last three to five years. Efficiency ratios and a few performance metrics are mentioned in the example instance. To prevent confusion, their computation and interpretation could be made clearer. 

 Overall Suggestion:

 This paper is timely and strong. The article will be scientifically sound and prepared for indexing with a few minor changes, especially in updating the literature base and making the interpretation clearer.

If applicable, is the statistical analysis and its interpretation appropriate?

Partly

Is the study design appropriate and is the work technically sound?

Yes

Is the work clearly and accurately presented and does it engage with the current literature?

Partly

Are the conclusions drawn adequately supported by the results?

Yes

Are sufficient details of methods and analysis provided to allow replication by others?

Yes

Are all the source data and materials underlying the results available?

Yes

Reviewer Expertise:

My research focuses on public administration, performance management, and policy evaluation in the context of regional and development planning.

I confirm that I have read this submission and believe that I have an appropriate level of expertise to confirm that it is of an acceptable scientific standard, however I have significant reservations, as outlined above.

## References

[1] : Performance measurement and management in the public sector: state of the art and research opportunities. *International Journal of Productivity and Performance Management* .2021;70(7) : 10.1108/IJPPM-03-2020-0102 1629-1654 10.1108/IJPPM-03-2020-0102

[2] : Export promotion programs: A system-based systematic review and agenda for future research. *Journal of World Business* .2022;57(4) : 10.1016/j.jwb.2022.101344 10.1016/j.jwb.2022.101344

# Response

**Published:** 2025-09-20

**Authors:** Stéphane Ruiz Coupeau

**Affiliations:** Universidad de Sevilla, Seville, Andalusia, Spain

We thank the reviewer for their encouraging and constructive review. Below, we outline how we have addressed the points for improvement.

*Comment:* Expand interaction with recent research in export promotion and public sector performance management (last 3–5 years).

*Response:*

 We have significantly enriched the manuscript’s literature review by incorporating recent sources (2021–2025), including:

- Bauer & Greiling (2024) on public sector sustainability controls
- Bovaird & Loeffler (2023) on public management and governance
- Choi et al. (2025) on EPAs during the pandemic
- Johnsen et al. (2024) on PMS design as a link between strategy and information use
- Jabbouri et al. (2022) on policy-practice decoupling

These additions strengthen the theoretical foundation and connect our findings with emerging research trends.

*Comment:* Clarify the computation and interpretation of ratios. *Response:* We revised the paragraph explaining the Key Performance Indicators to provide a more detailed and transparent account, explicitly listing and describing all KPIs within the text. In addition, we clarified the computation and interpretation of the outcome-per-resource efficiency ratios, ensuring that readers understand how these measures are derived and applied in practice. To avoid potential confusion, we removed cost-based ratios and retained only FTE-based indicators, which offer greater reliability and comparability across contexts.

# Reviewer report: Reviewer response for version 1

**DOI:** 10.21956/openreseurope.22230.r57609

**Published:** 2025-08-28

**Authors:** Beatriz García Cornejo, José Antonio Pérez Méndez

**Affiliations:** 1 Accounting, Universidad de Oviedo Facultad de Economia y Empresa (Ringgold ID: 83181), Oviedo, Asturias, Spain

Summary of the Article:

 The paper examines how Export Promotion Agencies (EPAs) can design and implement Performance Management Systems (PMSs) using the Logic Model as a structuring and flexible tool. Drawing on an exploratory multiple-case study of Spanish EPAs at provincial, regional, national, and European-network levels, the authors compare PMS maturity, describe practices linked to the model’s components (inputs, activities, outputs, outcomes), and propose a step-by-step methodology. An extended case from a European networked EPA illustrates a relatively mature PMS with client journeys, standardized KPIs, outcome follow-ups, and cost-per-outcome metrics. The contribution is primarily applied: a structured pathway to operationalize PMSs in EPAs and to strengthen learning, accountability, and service effectiveness.

 Overall Assessment:

 This is a clear, well-written, and timely contribution on a topic of high relevance for both professional practice and academic literature. The Logic Model is appropriately adapted to the EPA context, and the combination of a multiple-case study with a detailed illustrative case offers practical and conceptual value. The suggestions below are intended to further enhance clarity, analytical depth, and transferability, building on the solid foundations already established in the manuscript.

 Is the work clearly and accurately presented and does it engage with the current literature? → Partly

 The article is well-structured, with relevant references to EPAs and performance management. The literature review is solid, and with some additional context, its engagement with broader governance and institutional debates could be further enriched:

 If possible, include an early, concise definition or visual representation of the Logic Model (inputs, activities, outputs, outcomes, impacts), with a brief note on its known limitations (e.g., linearity, rigidity). This would make the framework more accessible to readers less familiar with it, without altering the paper’s scope.

 To help readers in other countries or institutional settings assess applicability, consider adding a short overview of the national EPA landscape (e.g., approximate number of agencies by governance level, typical staff size, and main categories of outcome indicators). Clarifying whether reporting on these indicators is mandatory or voluntary would also help explain differences in PMS adoption. If readily available data are lacking, even approximate figures or references to public reports would be sufficient.

 When discussing the challenges EPAs face in navigating stakeholder diversity, it could be useful to note that these external accountability pressures often come from multiple actors (e.g., ministries, EU funders, regional governments, business clients). Such multi-actor contexts may encourage alignment in some cases but can also create conflicting demands, potentially fostering decoupling — where performance indicators are collected mainly for compliance rather than for strategic management. Making this nuance explicit—already implicit in the findings—would add depth to the institutional analysis.

 Is the study design appropriate and is the work technically sound? → Yes

 The multiple-case study design is appropriate for the exploratory aim. The variation in agency type and governance context supports analytical generalization, and the extended European-network case provides a strong “high-maturity” example.

 Are sufficient details of methods and analysis provided to allow replication by others? → Partly

 The section on data collection and analysis is generally clear, including the description of triangulation and coding. To make the process even more transparent, it might be useful to briefly indicate whether any qualitative analysis software was used.

 It would also be helpful to clarify whether the extended case’s Logic Model (Figure 1) and the indicators in Table 2 were developed by the authors from interviews and operational data, or whether they follow a formalized framework required by the European network. This distinction would help readers understand the degree of author operationalization versus adherence to an existing standardized model.

 If the indicators in Table 2 are part of a standardized EU framework, noting whether complementary, context-specific indicators (e.g., regional economic impact, sector-specific export growth, employment effects) are allowed at the local node level would be informative.

 For Efficiency Ratios 2 and 4, it would help to clarify whether “Cost” covers the full budget for the specific service, only direct expenses, or the agency’s total operational expenditure. Without this definition, it is difficult for readers to determine whether these ratios indicate service-level cost-effectiveness, agency-wide productivity, or another type of efficiency measure.

 Additionally, if information is readily available **,** the following points could further enhance the paper’s value:

- The findings already reveal significant variability in PMS sophistication and Logic Model adoption, with some provincial and regional agencies relying more on informal approaches. Briefly noting how benchmarking across agencies—such as identifying common success factors or recurring implementation challenges—could support the transition toward more structured PMSs might enhance the paper’s practical relevance.
- Mentioning whether EPAs’ management systems incorporate sustainability dimensions (economic, social, environmental) might broaden the paper’s relevance.
- A simple comparative table of selected KPIs, ratios, or indicators could make differences in PMS formality and focus more tangible; if such data are not available, this could be signalled as a possible avenue for future research.

Are all the source data and materials underlying the results available? → Yes

 The availability of interview transcripts and supporting materials (e.g., via Zenodo) supports transparency.

 If applicable, is the statistical analysis and its interpretation appropriate? → Not applicable

 The study is qualitative; no statistical analysis is expected.

 Are the conclusions drawn adequately supported by the results? Partly

 The conclusions are consistent with the findings. They could be made even stronger by:

- Structuring them explicitly around the two research questions.
- Stating more clearly the specific conditions under which the proposed PMS is most feasible (e.g., stable funding, data infrastructure, internal capacity). Although these conditions are already present in the findings, making them more explicit would help readers see the circumstances under which the PMS is most likely to succeed.
- Providing a concrete example—possibly from the European network case—of how performance indicators have informed resource allocation or service redesign. This would illustrate the concept of “reduced decoupling” and reinforce the idea that PMSs can support learning and strategic adaptation.

In our view, the manuscript already makes a valuable contribution and is suitable for indexing once the suggested clarifications are considered. These points are intended to further highlight the strengths of the study and broaden its impact.

If applicable, is the statistical analysis and its interpretation appropriate?

Not applicable

Is the study design appropriate and is the work technically sound?

Yes

Is the work clearly and accurately presented and does it engage with the current literature?

Partly

Are the conclusions drawn adequately supported by the results?

Partly

Are sufficient details of methods and analysis provided to allow replication by others?

Partly

Are all the source data and materials underlying the results available?

Yes

Reviewer Expertise:

Management accounting, management and control systems in health care, financial and efficiency analysis, agricultural economics

We confirm that we have read this submission and believe that we have an appropriate level of expertise to confirm that it is of an acceptable scientific standard, however we have significant reservations, as outlined above.

# Response

**Published:** 2025-09-20

**Authors:** Stéphane Ruiz Coupeau

**Affiliations:** Universidad de Sevilla, Seville, Andalusia, Spain

We would like to thank the reviewers for their constructive and thoughtful comments. We have revised the manuscript accordingly to enhance analytical depth, transparency, and relevance. Below, we respond point-by-point to each suggestion.

*1. Comment:* Include a concise definition or visual representation of the Logic Model with a brief note on its limitations.

*Response:*

 We have included a visual representation of the Logic Model as Figure 1 in the revised version, immediately following the paragraph that defines the model. We have also added a brief discussion of its known limitations, including assumptions of linearity and the challenges in capturing intangible or emergent outcomes.

*2. Comment:* Add a short overview of the Spanish EPA landscape and clarify reporting obligations.

*Response:*

 We have rewritten the relevant part of the “Research Design” section to provide an overview of EPAs in Spain, their staff sizes, governance levels, and the nature of performance indicators.

*3. Comment:* Discuss multi-actor accountability and the risk of decoupling.

*Response:*

 We have added a paragraph at the end of the section “Export promotion agencies and their performance management needs” to explicitly discuss external accountability pressures, multi-actor dynamics, and the risk of symbolic compliance or decoupling.

*4. Comment:* Indicate whether software was used.

*Response:*

 We clarified in the “Data analysis” section that no qualitative analysis software was used. All coding and interpretation were performed manually.

*5. Comment:* Clarify whether Figure 1 and Table 2 follow a formal framework or are author-generated.

*Response:*

 We included a sentence noting that Figure 1 (Figure 2 in the new version) and Table 2 were developed by the authors by integrating interview findings and secondary data.

*6. Comment:* Note whether local indicators are allowed under the European network’s framework.

*Response:*

 We have added: The referenced system is standardized across the network, which does not permit the use of context-specific indicators at the local node level.

*7. Comment:* Explain whether “Cost” includes total or partial expenditures.

*Response:*

 This prompted us to reassess the use of efficiency ratios. We removed the cost-based indicators due to unreliability in cross-country comparisons and retained only FTE-based ratios, which are more robust and comparable.

*8. Comment:* Explain how benchmarking could support PMS evolution.

*Response:*

 A paragraph was added to the “Discussion” section, emphasizing the role of benchmarking in learning, convergence, and standardization of PMS practices, supported by relevant literature.

*9. Comment:* Discuss whether EPAs consider social and environmental performance.

*Response:*

 We added a new paragraph in the discussion section noting that current PMSs largely omit sustainability indicators, and explain how the Logic Model offers flexibility to integrate ESG and SDG-aligned measures.

*10. Comment:* Suggest creating a comparative table of indicators.

*Response:*

 We acknowledged the value of this suggestion and added a paragraph indicating that a comparative table of KPIs and efficiency indicators could be developed in future research

*11. Comment:* Structure conclusions explicitly around the research questions, conditions for PMS success, and include examples.

*Response:*

 We rewrote the conclusion to align directly with the two research questions and to include conditions for successful PMS implementation. We also added a concrete example from the European network agency, illustrating how indicators inform strategic decisions and reduce decoupling.
